# Winter Conditions and Land Cover Structure the Subnivium, A Seasonal Refuge beneath the Snow

**DOI:** 10.1371/journal.pone.0127613

**Published:** 2015-05-29

**Authors:** Sonia K. Petty, Benjamin Zuckerberg, Jonathan N. Pauli

**Affiliations:** Department of Forest and Wildlife Ecology, University of Wisconsin-Madison, Madison, Wisconsin, United States of America; Southern Illinois University, UNITED STATES

## Abstract

In seasonally snow-covered environments, many organisms endure winter by using the subnivium, a below-snow thermally stable seasonal refugium. Because the insulation of snow is dependent on snow depth and density, the stability of temperatures within the subnivium varies across land cover types. Additionally, across much of the Northern Hemisphere snow extent, depth and duration are generally decreasing while snow density is increasing due to climate change. These changes are likely to destabilize the thermal profile of the subnivium, although they have not yet been quantified. To explore the effects of land cover and climate change on the subnivium, we measured snow pack characteristics (depth and density), and ambient and subnivium temperatures from three different land cover types (prairie, deciduous forest, and coniferous forest) and within a micro-greenhouse (2.5 x 2.5 x 2 m) that maintained a temperature of 5°C warmer than outdoor ambient temperatures, and automatically opened during snow events throughout the winter of 2013/14. We found that the mean daily subnivium temperature was significantly colder in the deciduous cover type than the prairie cover type, and that prairie had higher maximum subnivium temperatures than both of the other cover types. Our climate change simulation revealed that, although ambient temperatures within the micro-greenhouse were 5°C warmer than outside the greenhouse, the daily minimum subnivium temperature was significantly lower inside the greenhouse. Our findings suggest that climate change could have considerable effects on the refuge quality of the subnivium, and that some cover types appear to be more susceptible to these effects than others.

## Introduction

Winter at high latitudes is a time of extreme environmental conditions and resource limitation, characterized by seasonal snow cover. During the height of the winter season, nearly half of the landmass in the Northern Hemisphere is covered in snow [1]. To survive winter, many overwintering organisms rely on less hostile and more thermally stable microclimates [2]. Underneath the snow exists an interface between the soil and snow, called the subnivium, which provides a thermally stable and predictable microhabitat [3]. The subnivium forms when heat is released from the soil and warm, moist air is trapped by the snow. As this air migrates through the snow pack it cools, and water vapor condenses, which creates a vertical gradient of increasing snow crystal size and a base layer of snow that is loose and granular [4]. Thus, the thermal stability of the subnivium is dependent on the insulative capacity of the snow, which increases with snow depth, but decreases with snow density [5].

Ultimately, the conditions within the subnivium are driven by ambient temperatures and snow characteristics, which are in turn strongly influenced by land cover type [6]. For example, the amount of canopy cover and ground vegetation mediate the balance of incoming and reflected radiation, as well as wind speed, and generally result in subnivium conditions in wooded areas being warmer, in some cases by 4–6°C, compared to open habitat types [7]. At the same time, however, as much as 40% of snowfall can be intercepted and sublimated in areas with dense canopy cover [8], delaying and reducing snow accumulation in heavily wooded areas [9]. Cover type not only modulates average subnivium temperatures, but also the variability and extremes of these temperatures, due to the timing and development of snow pack conditions. Even fine-scale differences in vegetation structure can strongly affect soil temperature and snow loss date; in fact, ground temperature measurements taken as little as 5 meters apart can vary by as much as 2.6°C [10]. Minimum and mean temperatures are lower in open habitats while maximum temperatures are higher [11], and forests in northern latitudes with intact canopies retain snow cover longer when mean temperatures are below -6°C [12]. Differences in understory vegetation yield similar results, with minimum and maximum temperatures becoming more extreme as the density of ground level vegetation decreases [6]. However, few studies have addressed how these effects change throughout the winter season, or the role that these differences play in maintaining the subnivium environment.

In addition to cover type, the characteristics of the subnivium are likely to be strongly influenced by climate change and associated changes in snow cover characteristics: notably, snow duration, depth and density. Where the snow pack exceeds 50 cm, soil surface temperatures are maintained near 0°C [13]. As snow depth decreases below 50 cm, however, the subnivium loses its insulating properties and soil surface temperatures decrease and become more variable—eventually tracking ambient temperatures. Between 1951 and 2012, global mean temperatures increased by 0.72°C [14], and during that time there have been strong negative trends in snow depth and snow cover extent in North America [15]. In the last 30 years, there has been a decrease in snow cover of 0.8 million km^2^ per decade [16]. As ambient temperatures increase, reduced snow depth and snow cover are likely to result in colder soil temperatures and increased freeze-thaw cycles [17,18]. Despite its ecological importance, the potential impact of climate change on the subnivium has not yet been explored, nor is it clear what role land cover type plays in maintaining subnivium quality in the face of warming winter conditions.

To quantify the effects of land cover type on subnivium temperature and stability, we recorded temperature, snow depth and density measurements at a prairie, deciduous forest, and coniferous forest sites. Ambient temperatures are generally warmer in wooded areas during the winter season [19], and such areas receive less solar radiation, which delays melting and enables the snow to persist throughout the winter [20]. However, wooded habitats with dense canopies and high snow interception rates accumulate less snow than open habitats, and we therefore predicted that coniferous forest cover type would feature the coldest and least stable subnivium temperatures. In contrast, because open prairie habitats accumulate greater quantities of snow at a faster rate, we predicted that prairies would have the most stable subnivium temperatures at the onset of winter. As winter progresses, and prairie habitats receive more incoming solar radiation and more snow is lost due to melting than other cover types [21], we predicted that the subnivium would become more thermally variable at the end of winter. To investigate the effects of climate change on the subnivium, we used a micro-greenhouse that maintained a temperature of 5°C warmer-than-outside and recorded temperature, snow depth and snow density measurements inside and outside of the micro-greenhouse throughout the winter of 2013–2014. Within the greenhouse, we predicted that the subnivium would be colder and more variable, especially at the end of the winter season.

### Study Area

We obtained permission from the University of Wisconsin-Madison to conduct our research in the UW Arboretum, roughly 485 hectares of restored habitat in south-central Wisconsin. Here, winters are characterized by cold temperatures (average daily mean temperature = -6.1°C) and moderate amounts of annual snowfall (average snow depth = 127.5 cm; Wisconsin State Climatology Office). We selected three different sites representative of tall grass prairie (Kentucky [*Poa pratensis*] and big bluegrass [*P*. *ampla*], and Canada goldenrod [*Solidago canadensis*], deciduous (mainly oaks [*Quercus* spp.]) and coniferous (white [*Pinus strobus*] and red pines [*P*. *resinosa*]) forest habitat.

## Methods

### Temperature and snow measurement

During the winter of 2013–2014, we collected subnivium and ambient hourly temperatures from each of our three sites (deciduous forest [UTM Z16N, 301334E 4767977N], coniferous forest [301114E 4767466N], and prairie [301991E 4768093N]; S1 Fig) using temperature data loggers (iButtons Maxim DS1922L-F5). Four ambient data loggers were deployed at each site, distributed at a distance of 5 m in each cardinal direction from a pre-determined center point. A subnivium temperature data logger was secured to the ground directly below each ambient data logger. Prior to the winter’s first snowfall, we placed data loggers inside 6.4-cm sections of PVC pipe and secured horizontally to the ground to measure subnivium conditions. The open ends of the PVC pipe were covered in wire mesh for ventilation while preventing the data loggers from coming into direct contact with the snow. We placed ambient temperature data loggers inside 6.4-cm pieces of PVC pipe that were suspended vertically from 1.2-meter tall metal poles (S2 Fig). The bottom of each PVC capsule was covered with wire mesh and a 10.2-cm diameter cap was elevated above the top of each capsule to prevent snow and other debris from falling in, while still allowing air to circulate. Each capsule was then coated with aluminum foil to minimize radiative exchanges.

From each site, we collected data on habitat characteristics we hypothesized might influence snow accumulation and alter ambient and subnivium conditions. Specifically, we measured the diameter at breast height (DBH; cm), counted the total number of trees, and estimated the height (m) of trees ≥ 8 cm DBH. In addition, we measured canopy closure at each site by quantifying percent transmittance using a plant canopy analyzer (LI-COR, LAI-2000). To understand whether our study plot was representative of the cover types—prairie, deciduous, and coniferous—we also collected these habitat characteristics from 4 random plots within each of the three general cover types.

To quantify the effects of future climate change on the subnivium, we utilized a micro-greenhouse (2.5 x 2.5 x 2 m) located in the University of Wisconsin-Madison Arboretum (S1 Fig). The micro-greenhouse had an automated roof that opened during precipitation and snow events, allowing the same amount of snow to accumulate inside and outside of the greenhouse. Once the precipitation event ended, the roof would close and the greenhouse would heat to a temperature of 5°C warmer than outside ambient temperatures. We selected this setting using mid-21^st^ century projections (2046–2065) to calculate the differences between current and future winter climate (December, January, and February). Future climate projections were based on the high emission scenario (A2) using an average of nine global climate models from the Coupled Model Intercomparison Project Phase 4 (CMIP4). The A2 family of scenarios generally reflects fewer regulations on world-wide emissions, increasing human populations, and regionally oriented economic development [22]; recent simulations suggest that warming by the mid-21^st^ century is increasingly consistent with this family of greenhouse-gas emissions scenarios [23].

We deployed four subnivium and ambient temperature data loggers inside and directly outside of the greenhouse, using the same arrangement as with our three cover types. All of the data loggers were collected on 28 March 2014, after the snow pack had melted.

In addition to temperature measurements, we recorded bi-weekly snow depth and snow density measurements. Snow depth was measured at the location of each temperature data logger using a meter stick and the average of these four measurements was calculated to represent the overall depth at each site. The snow density (or sink depth) at each site was measured using a cylindrical snow penetrometer.

### Statistical Analysis

We used a multivariate analysis of variance (MANOVA) to compare the habitat characteristics of our sampling plots to the randomized plots within the same cover type for deciduous and coniferous (we did not analyze measurements from the prairie habitat because they were structurally homogeneous). Specifically, we used MANOVA to compare the multiple habitat variables (i.e., number of trees, DBH, tree height, and transmittance) within and between our cover types. For each sensor, we calculated four averaged temperature metrics (°C): daily mean (MEAN), daily minimum (MIN), daily maximum (MAX), and daily variance (VAR). We used generalized liner mixed effects models (GLMM) to quantify the differences between subnivium and ambient temperature profiles between cover types. The four temperature metrics represented separate response variables while cover type (deciduous, coniferous, prairie) were modeled as fixed effects. Due to the repeated nature of the temperature data, we included data logger ID as a random effect. By including this random effect, we take into account random variation in the individual data loggers, eliminate artifacts of pseudoreplication and account for spatial variation between data loggers within plots. All models were fit using a Gaussian error distribution, and we performed residual analysis on model errors. We implemented the GLMM model using the R statistical platform [24], using the lme4 package. We used lmerTest for assessing contrast effects and estimating differences in least squares means (population means) [25]. To visualize changes in the subnivium environment, we fit a generalized additive mixed model (GAMM) with data logger included as a random effect and daily mean daily temperature as the response variable [26]. We assumed an *a priori* significance level of α = 0.05.

## Results

**Fig 1:**
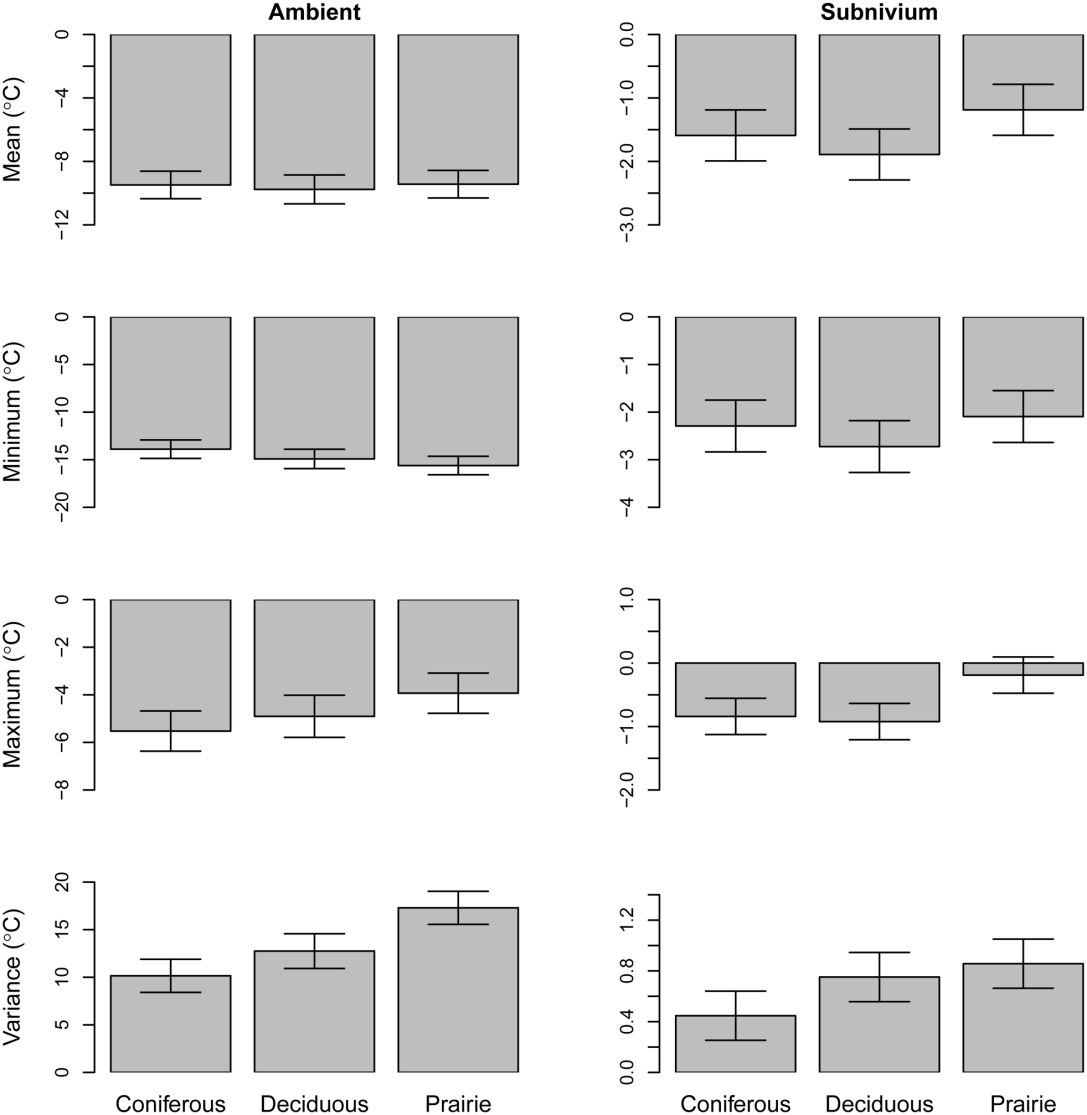
Subnivium Temperatures Across Different Cover Types. Differences in the daily mean, minimum, maximum, and variance winter temperatures (°C) across the three land cover types (coniferous, deciduous, and prairie). For each cover type we present the least square means and 95% confidence intervals for ambient (left column) and subnivium (right column) temperature profiles.

Our study plot within the deciduous forest type was not significantly different compared to random plots within the same cover type in terms of number of trees (F_1,26_ = 0.0269, *P* = 0.87), DBH (F_1,26_ = 1.77, *P* = 0.20), tree height (F_1,26_ = 2.29, *P* = 0.14), and transmittance (F_1,26_ = 3.79, *P* = 0.06). Similarly, within the coniferous site, our study plots did not differ from random plots in number of trees (F_1,23_ = 0.08, *P* = 0.783), DBH (F_1,23_ = 0.72, *P* = 0.40), tree height (F_1,23_ = 1.23, *P* = 0.28), or transmittance (F_1,23_ = 3.84, *P* = 0.06). Across cover types, compared to deciduous and coniferous sites, the prairie site was characterized by significantly fewer trees (F_2,55_ = 8.72, *P* < 0.001) and higher transmittance (F_2,55_ = 371.8, *P* < 0.001). There was no significant difference in the number of trees between deciduous and coniferous sites (F_1,51_ = 8.72, *P* = 0.69), but the coniferous sites had trees of a significantly higher height (F_1,51_ = 35.4, *P* < 0.001) and DBH (F_1,51_ = 13.65, *P* < 0.001), and lower transmittance (F_1,51_ = 151.6, *P* < 0.001). Throughout the months of December and January, there were no significant differences in mean ambient temperature between any of the cover types (*β*_MEAN_ = 0.30, SE = 0.64, *P* > 0.05) (Fig 1). Comparing the differences of least squares means between cover types, the coniferous site had significantly higher minimum (*β*_MIN_ = 1.70, SE = 0.70, *P* = 0.01) and lower maximum (*β*_MAX_ = -1.60, SE = 0.61, *P* < 0.01) ambient temperatures than the prairie site (Fig 1), but there were no difference in these same metrics between coniferous vs. deciduous (*β*_MIN_ = 1.0, SE = 0.72, *P* > 0.05; *β*_MAX_ = -0.60, SE = 0.62, *P* > 0.05) or deciduous vs. prairie cover types (*β*_MIN_ = 0.70, SE = 0.72, *P* > 0.5; *β*_MAX_ = -1.00, SE = 0.62, *P* > 0.05). All three sites had differences in the variability of ambient temperatures; prairie sites were the most variable followed by deciduous and then coniferous forest. The ambient temperatures in the coniferous forest varied less than in the deciduous forest (*β*_VAR_ = -2.60, SE = 1.28, *P* = 0.04), and both coniferous and deciduous forest varied less compared to prairie (*β*_VAR_ = -7.10, SE = 1.25, *P* < 0.001; *β*_VAR_ = -4.50, SE = 1.28, *P* < 0.001) (Fig 1).

In contrast to the average mean ambient temperature, which did not vary by cover type, the average mean subnivium temperature in our deciduous site was colder than the prairie site (*β*_MEAN_ = -0.70, SE = 0.26, *P* = 0.02) (Fig 1). There was no such difference between coniferous vs. prairie (*β*_MEAN_ = -0.40, SE = 0.26, *P* > 0.10) or coniferous vs. deciduous cover (*β*_MEAN_ = 0.30, SE = 0.26, *P* > 0.10). However, temperature profiles of daily mean subnivium temperature showed that, similar to the deciduous cover type, the coniferous cover type remained below 0°C longer into the spring season than the prairie cover type. The average maximum subnivium temperature was also higher in the prairie site than in both the coniferous and deciduous sites (*β*_MAX_ = -0.70, SE = 0.19, *P* < 0.01; *β*_MAX_ = -0.70, SE = 0.19, *P* < 0.01), with no difference between the coniferous vs. deciduous cover types (*β*_MAX_ = 0.10, SE = 0.19, *P* > 0.10) (Fig 1). There was also no difference in average minimum subnivium temperature between the three cover types (*β*_MIN_ = -0.6, SE = 0.35, *P* > 0.10; *β*_MIN_ = -0.20, SE = 0.35, *P* > 0.10; *β*_MIN_ = 0.40, SE = 0.35, *P* > 0.10). However, average daily variance in subnivium temperature differenced among the sites, with both prairie and deciduous having greater variance in subnivium temperature than the coniferous site (*β*_VAR_ = -0.40, SE = 0.14, *P* < 0.01; *β*_VAR_ = -0.30, SE = 0.14, *P* < 0.03) (Fig 1). There was no significant difference in subnivium temperature variance between the prairie vs. deciduous cover types (*β*_VAR_ = -0.10, SE = 0.14, *P* > 0.10).

**Fig 2:**
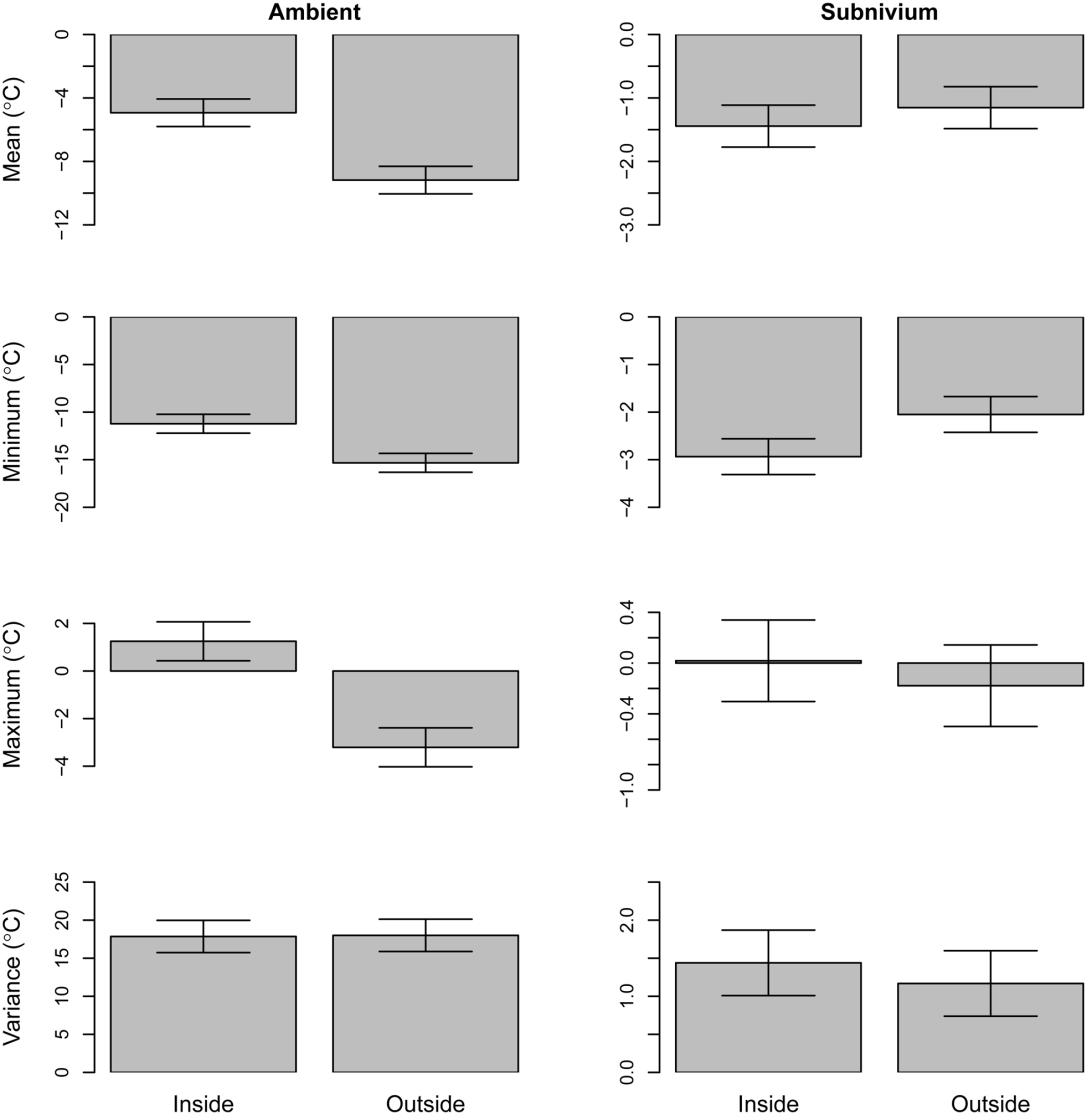
Subnivium Temperatures Within and Outside the Experimental Greenhouse. Differences in the daily mean, minimum, maximum, and variance using winter temperatures (°C) within and outside the experimental greenhouse. We used least square means and 95% confidence intervals for ambient (left column) and subnivium (right column) temperature profiles.

Within the greenhouse the average daily mean, maximum and minimum ambient temperatures were all significantly higher compared to outside of the greenhouse (*β*_MEAN_ = 4.20, SE = 0.63, *P* < 0.01; *β*_MAX_ = 4.50, SE = 0.59, *P* < 0.01; *β*_MIN_ = 4.10, SE = 0.72, *P* < 0.01) (Fig 2), while there was no difference in ambient temperature variance between the two sites (*β*_VAR_ = -0.10, SE = 1.53, *P* > 0.10). There were no differences in average daily mean and maximum subnivium temperatures inside vs. outside the greenhouse, nor in average daily subnivium temperature variance (*β*_MEAN_ = -0.30, SE = 0.24, *P* > 0.10; *β*_MAX_ = 0.20, SE = 0.23, *P* > 0.10; *β*_VAR_ = 0.3, SE = 0.31, *P* > 0.10) (Fig 2). However, despite the minimum ambient temperature in the greenhouse being higher than outside the greenhouse, the minimum subnivium temperature within the greenhouse was significantly lower than that outside (*β*_MIN_ = -0.90, SE = 0.27, *P* < 0.01) (Fig 2).

**Fig 3:**
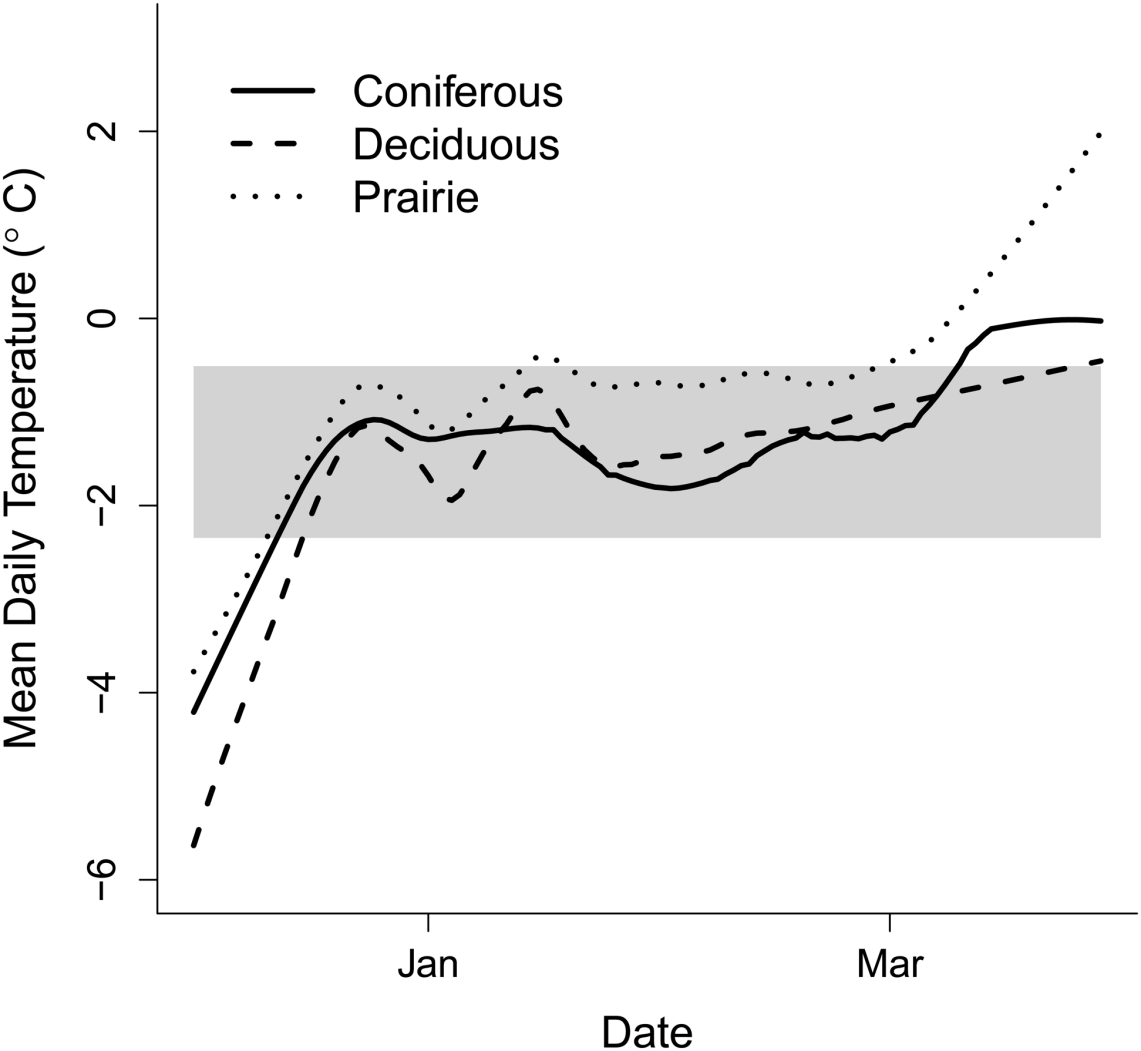
Temperature profiles of daily mean subnivium temperature (°C) across the three land cover types. The profiles are created using the generalized additive mixed model. The gray box represents the 1^st^ and 3^rd^ quartile of the winter mean temperature range for all sites.

**Fig 4:**
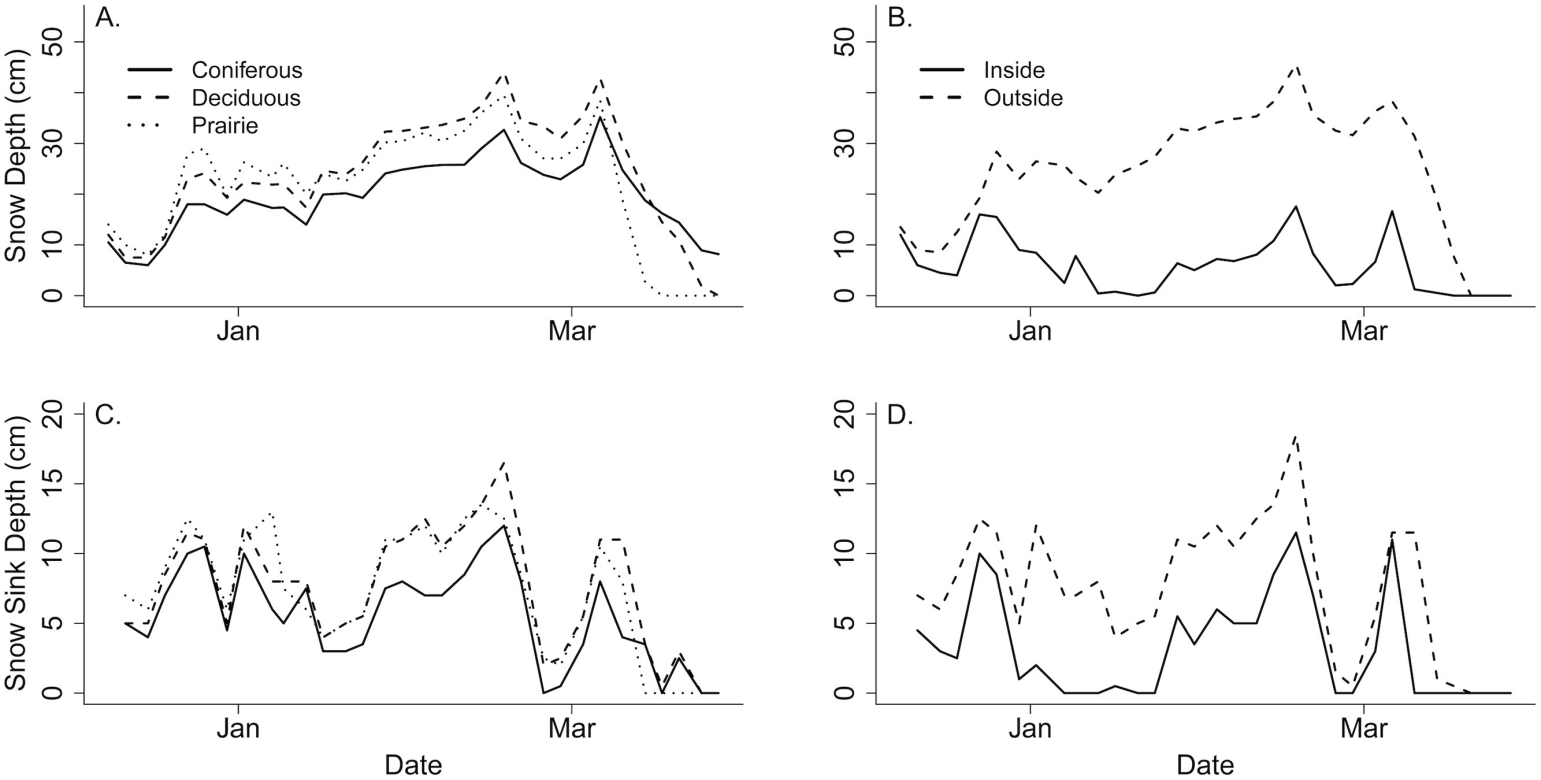
Snow Depth and Density Measurements. Snow depth (cm) and sink depth (cm, or density) measurements taken from coniferous, deciduous and praire cover types (A, C), and inside vs. outside the microgreenhouse (B, D), throughout the winter of 2013–2014.

The deciduous cover type was the coldest at the onset of winter, and took the longest to stabilize (Fig 3), while the prairie site was consistently warmer and broke free from the subnivium earlier in the spring season. Prairie accumulated snow more rapidly at the onset of winter compared to the other cover types, and also lost the snow cover faster at the onset of spring (Fig 4). In contrast the coniferous forest accumulated snow the slowest, but maintained snow cover longer into the spring season than the other two cover types. The coniferous forest also had a denser snow cover compared to the other cover types. Snow measurements taken inside and outside of the greenhouse also showed noticeable differences, with snow depth being significantly lower and more variable inside of the greenhouse (Fig 4). Similarly, the snow was consistently denser inside the greenhouse than outside, and there was a brief period (2–3 days) in January when there was no snow inside the greenhouse.

## Discussion

Our findings reinforce the concept that habitat type can have a strong influence on ambient conditions, leading to significant differences in subnivium conditions. We observed higher maximum ambient temperatures and lower minimum ambient temperatures in our open (prairie) cover type compared to the two forest sites, although we did not detect any difference in mean ambient temperature between the three cover types. Interestingly, despite the lack of difference in mean ambient temperature, we found that the deciduous forest possessed a subnivium with the lowest mean temperatures, and that the subnivium in both the deciduous forest and prairie cover types possessed the most variation in temperature. In contrast to our findings, previous studies had found that subnivium conditions were warmer in wooded areas compared to those in open areas [7]. Differences in snow depth and density may account for these inconsistencies, as the deciduous forest had less snow accumulation and a denser snow cover than the prairie, but colder ambient temperatures than the coniferous (Fig 4). In addition, the prairie cover type reached warm, stable subnivium temperatures much earlier in the winter season than the other two cover types, and showed a more rapid increase in temperature at the onset of spring (Fig 3).

Our climate change simulation showed that, despite ambient temperatures inside the greenhouse being an average of 4–5°C warmer than outside, the minimum subnivium temperatures were significantly colder than outside of the greenhouse. Higher temperatures inside the greenhouse increased the rate of snow melt, resulting in a thinner, denser snow cover with reduced insulative capacity (Fig 4). While the effects of climate change are likely to be less pronounced during the middle of winter when ambient temperatures are significantly below the 0°C melting point, there may be more drastic effects during the early winter and late winter periods, when ambient temperatures are higher and more variable, and the snow is more vulnerable to melting.

Colder subnivium temperatures and more frequent freeze-thaw cycles could have important implications for a variety of overwintering organisms including microbes, freeze-tolerant invertebrates and herptiles, and hibernating mammals [3]. How the distribution of the subnivium affects the fine-scale distribution of these species, and how deterioration of the subnivium from climate change will affect species distributions on a larger scale is difficult to predict. Habitat selection is influenced by a wide variety of factors ranging from temperature and humidity to vegetation structure, so it is possible that overwintering species will actively select for microhabitats in which the subnivium conditions are most suited for their energetic needs, creating small shifts in distribution even within the same cover type. Of the three habitats used in our study, deciduous forest had the coldest subnivium conditions, suggesting that species capable of shifting habitat use could potentially benefit from selecting different habitats (in this case prairie or coniferous) for overwintering purposes. On a larger scale, some populations may shift poleward or towards higher latitudes tracking suitable snow conditions, as has been observed in other studies [27, 28, 29]. However, species that are less mobile or have more restricted ecological niches may experience a retraction in range, and are the most vulnerable to altered winter conditions [30, 31]. In order to effectively manage species in the face of climate change, it is important that we better understand not only how the deteriorating subnivium will affect species survival on an individual level, but also how this will affect the distribution and interaction between multiple species on a larger scale. Further research on species responses to the characteristics of the subnivium, and how these responses might vary spatially and temporally are needed.

## Supporting Information

S1 Data Temperature and vegetation data measured during the winters of 2013/2014 and 2014/2015, respectively. (ZIP)

S1 Fig Study area and site location, featuring major cover types (National Landcover Dataset 2011), at the University of Wisconsin-Madison Arboretum. (TIF)

S2 Fig Temperature Data Logger Capsule and Micro-Greenhouse. a) PVC capsule built to house ambient temperature data loggers (iButtons Maxim DS1922L-F5). Each capsule consisted of a 6.4-cm section of PVC pipe suspended vertically from a 1.2-meter long metal pole. The bottom of the capsule was covered with wire mesh on which the data logger rested, while a 10.2-cm diameter PVC cap was elevated above the top of the capsule to protect from precipitation and falling debris. The PVC was then coated with aluminum foil to minimize radiative exchanges. b) Microgreenhouse (2.5 x 2.5 x 2 m) utilized to simulate future projected winter temperatures resulting from climate change. The automated roof opened during precipitation events allowing snow to accumulate within the greenhouse; once closed, the greenhouse heated to 5°C warmer than the outside air.
(TIF)
