# Supplementary figures and images for: Winter Conditions and Land Cover Structure the Subnivium, A Seasonal Refuge beneath the Snow
Source: PLoS One. 2015 May 29;10(5):e0127613. doi: 10.1371/journal.pone.0127613 (PMC4449108; doi:10.1371/journal.pone.0127613)

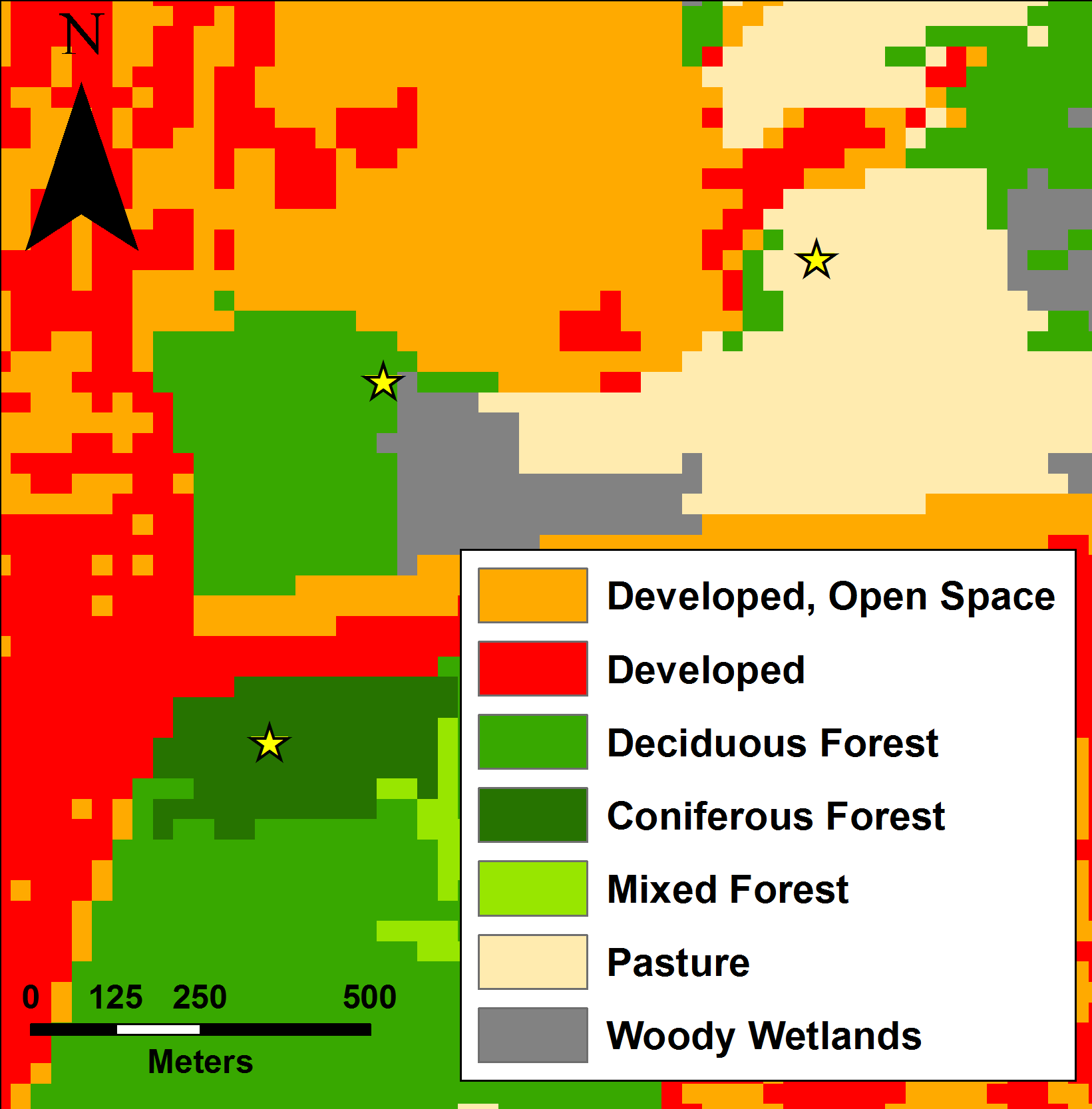

Supplement: S1 Fig — (TIF) [file pone.0127613.s002.tif]

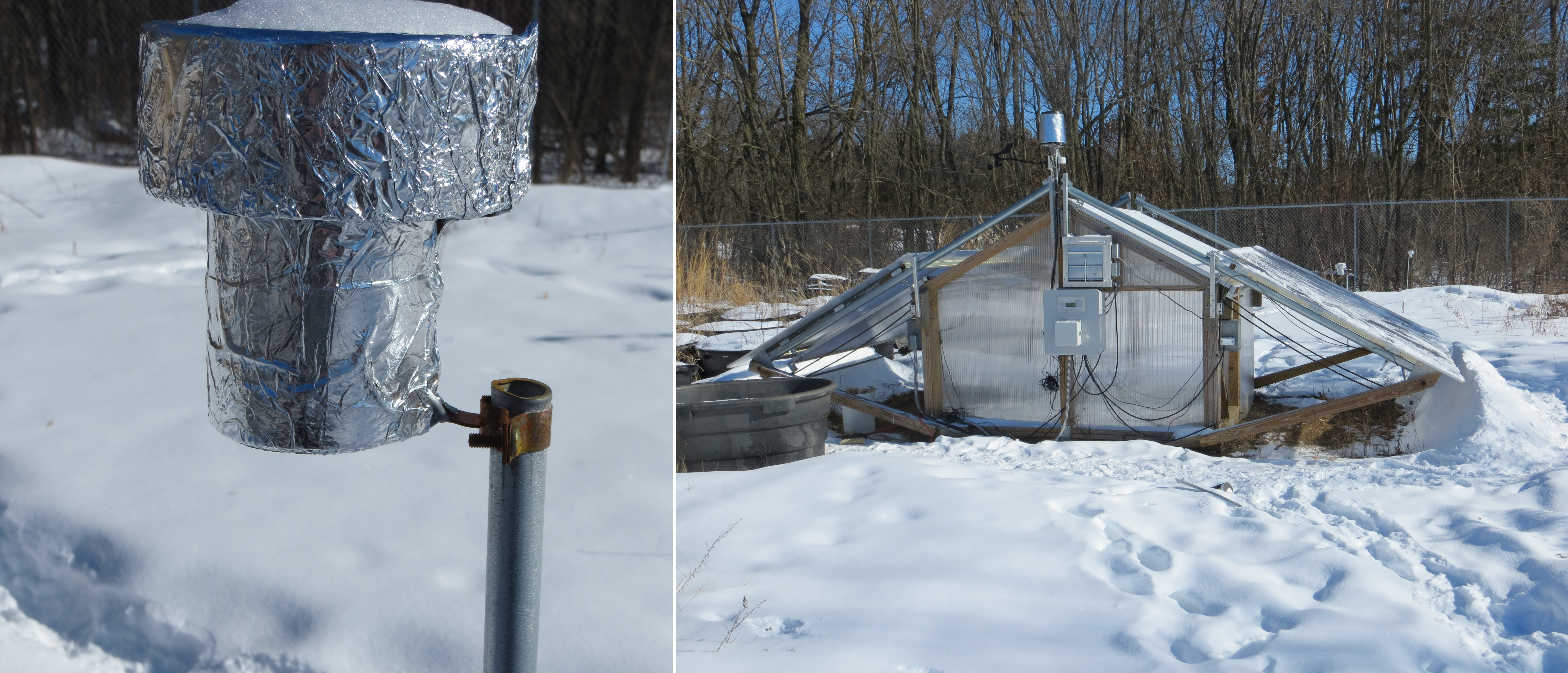

Supplement: S2 Fig — a) PVC capsule built to house ambient temperature data loggers (iButtons Maxim DS1922L-F5). Each capsule consisted of a 6.4-cm section of PVC pipe suspended vertically from a 1.2-meter long metal pole. The bottom of the capsule was covered with wire mesh on which the data logger rested, while a 10.2-cm diameter PVC cap was elevated above the top of the capsule to protect from precipitation and falling debris. The PVC was then coated with aluminum foil to minimize radiative exchanges. b) Microgreenhouse (2.5 x 2.5 x 2 m) utilized to simulate future projected winter temperatures resulting from climate change. The automated roof opened during precipitation events allowing snow to accumulate within the greenhouse; once closed, the greenhouse heated to 5°C warmer than the outside air. (TIF) [file pone.0127613.s003.tif]
